# Research on multimodal dense small object detection algorithms guided by cross-modal information

**DOI:** 10.1371/journal.pone.0348533

**Published:** 2026-09-08

**Authors:** Jiacheng Hu, Yumeng Ma, Yue Xing, Yun Zi, Yingnan Deng, Ming Wang, Heyao Liu, Junliang Du

**Affiliations:** 1 A. B. Freeman School of Business, Tulane University, New Orleans, United States of America; 2 Ira A. Fulton School of Engineering, Arizona State University, Tempe, United States of America; 3 School of Engineering and Applied Science, University of Pennsylvania, Philadelphia, United States of America; 4 School of Computer Science, Georgia Institute of Technology, Atlanta, United States of America; 5 College of Graduate and Professional Studies, Trine University, Phoenix, United States of America; 6 College of Engineering, Northeastern University, Boston, United States of America; 7 MoE Key Lab of Artificial Intelligence, AI Institute, Shanghai Jiao Tong University, Shanghai, China; PLOS: Public Library of Science, UNITED KINGDOM OF GREAT BRITAIN AND NORTHERN IRELAND

## Abstract

To address the challenges of modality heterogeneity, scale inconsistency, and background interference in dense small object detection under multimodal conditions, this paper proposes a novel detection framework based on cross-modality guidance and hierarchical scale refinement. Built upon the RT-DETR backbone, the framework integrates a Cross-Modality Guided Dynamic Fusion (CMG-DF) module, which performs semantic-level recalibration between infrared and visible features via a learnable modality attention mechanism, and a Hierarchical Scale Refinement Network (HSRN), which enhances semantic consistency and boundary continuity across scales through bidirectional residual flow and graph-based relational modeling. To validate the effectiveness of the proposed method, extensive comparison and ablation studies are conducted on two public multimodal benchmarks, SMOD and LLVIP. Experimental results show that the proposed method achieves 92.7% mAP@50 and 69.5% mAP@50:95 on SMOD, as well as 77.3% mAP@50 and 43.1% mAP@50:95 on LLVIP, consistently outperforming existing state-of-the-art multimodal detection algorithms. Qualitative visualizations further confirm the robustness and enhancement capability of the method for small objects under low illumination, occlusion, and complex background conditions, highlighting its strong structural generalization and practical deployment potential.

## 1 Introduction

In recent years, with the rapid advancement of multimodal perception technologies, object detection methods that integrate visible and infrared imagery have shown great potential in various applications such as intelligent surveillance, autonomous driving, and low-light imaging [1]. Particularly in nighttime, occluded, or complex illumination scenarios, single-modality approaches often suffer from issues such as missing information, blurred boundaries, and strong background interference. These limitations have motivated researchers to explore cross-modal collaborative modeling strategies to achieve more robust perception capabilities [2]. Meanwhile, dense small objects—characterized by limited size, weak texture features, and imbalanced scale distributions—have emerged as one of the most challenging problems in multimodal object detection. Therefore, how to achieve efficient cross-modal feature alignment and semantic enhancement for small objects remains an open and critical challenge in this field [3].

Although prior studies have made notable progress in multimodal fusion strategies and small object perception mechanisms, existing methods still face two key bottlenecks. First, the heterogeneity between modalities leads to difficulties in feature alignment, making it challenging to produce semantically consistent responses during fusion [4]. Second, small objects are prone to information loss and boundary degradation during deep semantic abstraction, especially in dense scenes where they are easily overwhelmed by background noise [5,6]. Moreover, many existing approaches rely on static strategies for cross-scale modeling and semantic focusing, lacking dynamic adaptability to varying object saliency across spatial hierarchies, which limits their generalization in real-world complex environments [7]. Beyond these issues, two remaining challenges are particularly critical for multimodal dense small-object detection: (i) cross-modal conflict and uncertainty, where modality-specific noise can dominate fusion and cause inconsistent responses on weak targets; and (ii) saliency imbalance across scales, where small objects exhibit highly non-uniform prominence at different feature levels, making it difficult for static fusion and fixed-scale enhancement to reliably preserve discriminative cues. To address these challenges beyond existing approaches, cross-modal information guidance should not merely aggregate features, but explicitly select mutually consistent cues while suppressing modality-conflicting signals, and further propagate the enhanced target evidence across scales to prevent small-object cues from being diluted during deep abstraction.

To address these challenges, we propose an end-to-end detection framework for multimodal dense small object detection. Our approach introduces a cross-modality guided mechanism and a hierarchical scale refinement structure, enabling dynamic modality fusion, semantic compensation, and scale-aware modeling. This design effectively resolves the difficulties of heterogeneous feature alignment and enhances the detection of small objects under multimodal conditions. Specifically, our cross-modal information guidance dynamically assigns modality-aware importance based on cross-modal consistency, allowing the network to emphasize complementary target cues while down-weighting conflicting background responses. Meanwhile, the hierarchical refinement stage redistributes the guided evidence across feature hierarchies, strengthening small-object saliency and localization fidelity in dense scenes where target cues are easily submerged. Built upon the mainstream RT-DETR architecture, the proposed model ensures high structural compatibility and inference efficiency, achieving robust small object recognition while maintaining lightweight computation.

The main contributions of this paper can be summarized as follows:

- We propose a cross-modality guided dynamic fusion module, which utilizes a learnable modality attention mechanism to achieve intra-feature semantic alignment and enhancement, effectively mitigating redundancy and conflict during multimodal fusion.
- We design a hierarchical scale refinement network that incorporates bidirectional residual paths and graph-based relational modeling to reinforce structural consistency across scales and maintain semantic continuity for small objects.
- We conduct systematic empirical studies on two public multimodal detection benchmarks, SMOD and LLVIP, demonstrating that our method consistently outperforms existing state-of-the-art approaches in terms of accuracy, stability, and interpretability, with strong potential for generalization.

## 2 Related work

### 2.1 Research on multimodal target detection methods

Recent years have witnessed remarkable progress in multimodal object detection across various applications such as remote sensing imagery, autonomous driving, and UAV-based perception [8–10]. In the remote sensing community, multimodal representation learning has also been extensively studied for classification and scene understanding, providing a methodological foundation for detection-oriented fusion designs [11]. Chen et al. [12] proposed a probabilistic ensembling framework for multimodal object detection, which jointly models candidate predictions from different modalities to enhance robustness and detection accuracy. Sharma et al. [13] introduced the YOLOrs framework to address multimodal fusion in remote sensing, constructing a shared semantic space between infrared and visible images to achieve stable detection of small objects. To further improve detail representation of small targets in low-resolution multimodal imagery, Zhang et al. [14] integrated super-resolution techniques into the proposed SuperYOLO architecture, demonstrating superior performance under limited resolution conditions. Meanwhile, the availability of large-scale multimodal benchmarks has further promoted rigorous evaluation under distribution shifts and complex scenes [15].

In terms of multimodal feature representation and fusion mechanisms, Xu et al. [16] proposed FusionPainting, which leverages adaptive attention to dynamically guide the fusion of heterogeneous modality features, significantly improving boundary modeling capabilities. Yuan et al. [17] proposed CMMDL, a cross-modal multi-domain learning framework for image fusion, highlighting the importance of robust fusion under modality and domain variations. Wu et al. [18] designed a virtual sparse convolution module to resolve structural misalignment between sparse LiDAR point clouds and image modalities, enabling structural adaptation across modalities. Valverde et al. [19] explored cross-modal distillation, where the auditory modality guides the visual stream under weak supervision, showing the potential of cross-modal learning in weakly labeled scenarios.

Overall, the research in multimodal object detection has evolved from traditional early-fusion approaches to deep fusion strategies centered around Transformer architectures, attention mechanisms, and high-order semantic modeling. Closely related progress has also been made in cross-modal tracking and RGBT tracking, where temporal association and cross-modal correspondence modeling are crucial for robust perception under challenging conditions [20,21]. Recent works by Zang et al. [22] and Wu et al. [23] have introduced large language models (LLMs) to provide contextual guidance for image region semantics and facilitate cross-modal alignment, offering a novel paradigm for multimodal small object detection. Despite these advances, achieving high-precision detection under challenges such as modality heterogeneity, resolution discrepancy, and densely packed objects remains a central problem in current research.

### 2.2 Progress in small target detection technology

Small object detection has long been a critical research topic in computer vision, with widespread applications in scenarios such as infrared imaging, remote sensing surveillance, and intelligent transportation. Zhao et al. [24] conducted a comprehensive review of single-frame infrared small object detection methods, highlighting the limitations of traditional approaches in suppressing background noise and identifying deep learning as the prevailing paradigm. To address the weak response of small targets in infrared images, Hou et al. [25] proposed RISTDNet, which incorporates a robust multi-scale residual module to enhance detection performance. Ma et al. [26] further designed an orthogonal information perception fusion (OIPF) framework, which effectively integrates multi-source features while preserving structural consistency, significantly improving target response in small regions.

To tackle the challenges of complex backgrounds and varying object scales, various enhancement strategies have been explored. Liu et al. [27], in their CVPR work, developed an infrared detection network with scale and position sensitivity, effectively mitigating the issue of small targets being overwhelmed by background clutter. Yang et al. [28] introduced a multi-attention mechanism into the Faster R-CNN framework to achieve more accurate perception in 6G-enabled intelligent transportation systems. In industrial defect inspection, Yue et al. [29] improved the YOLO architecture to better detect subtle flaws in fabrics, thereby enhancing both accuracy and robustness. Wu et al. [30] combined local fully convolutional networks with YOLOv5 for remote sensing-based small object detection, demonstrating the feasibility of lightweight networks for real-world deployment.

Overall, the evolution of small object detection has progressed from shallow architectures to high-performance frameworks centered on attention mechanisms, multi-scale feature enhancement, and deep perceptual modeling. Zang et al. [31] proposed DCANet, which leverages dense convolutional attention to improve local feature extraction for infrared small targets. Sun et al. [32] explored the application of the latest YOLOv8 architecture in surveillance scenarios for intelligent detection of fine-grained targets. Liu et al. [33] provided a survey on video-based small object detection methods, emphasizing the significance of temporal modeling and background suppression in dynamic environments. Collectively, these studies have advanced the practicality and intelligence of small object detection in multimodal, dynamic, and weak-signal scenarios.

## 3 Method

**Fig 1:**
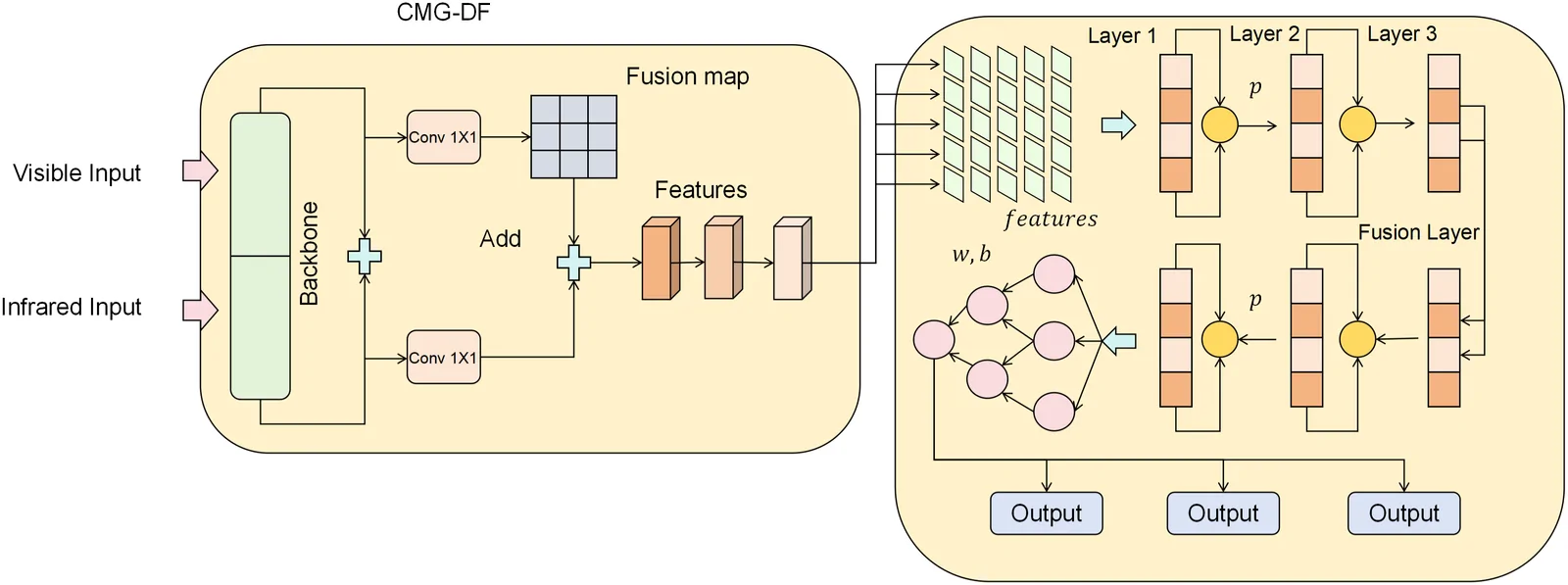
The figure shows the proposed multimodal dense small target detection framework: the CMG-DF module on the left generates a multi-scale feature sequence by dynamically fusing visible light and infrared features across modalities. The hierarchical scale progressive refinement network on the right refines the fused features layer by layer and outputs small target detection results of different scales in the multi-level detection head.

This paper addresses the dual challenges posed by scale variation and modality discrepancy in detecting dense small objects under complex backgrounds. We propose an end-to-end detection framework that integrates two core innovations. First, we introduce a Cross-Modality Guided Dynamic Fusion module, which employs learnable cross-modal attention weights to perform spatiotemporal dynamic recalibration of visible and infrared features, thereby mitigating semantic misalignment caused by modality inconsistency at the source level. Second, we develop a Hierarchical Scale Refinement Network, which leverages cascaded multi-resolution context aggregation and feature resampling to progressively enhance small object saliency while preserving fine-grained boundary details in densely populated scenes. The synergy of these two components enables the model to achieve more accurate and robust detection and localization of weak and small targets across heterogeneous modalities, while maintaining computational efficiency. The overall model architecture is shown in Fig 1.

Different from conventional multimodal fusion strategies that mainly rely on direct concatenation, summation, or static attention-based recalibration, the proposed CMG-DF explicitly models modality-dependent reliability and dynamically adjusts the contribution of visible and infrared features according to cross-modal semantic consistency. This design is particularly important for dense small target detection, where weak targets are easily affected by illumination changes, background clutter, and modality-specific noise. In addition, HSRN is not a simple multi-scale feature aggregation structure; it introduces hierarchical residual propagation and scale-aware refinement to preserve small-object boundary cues while enhancing semantic consistency across feature levels. Therefore, the two modules are designed to address the specific challenges of multimodal dense small target detection rather than merely combining existing fusion and multi-scale enhancement operations.

### 3.1 Cross-modality guided dynamic fusion

**Fig 2:**
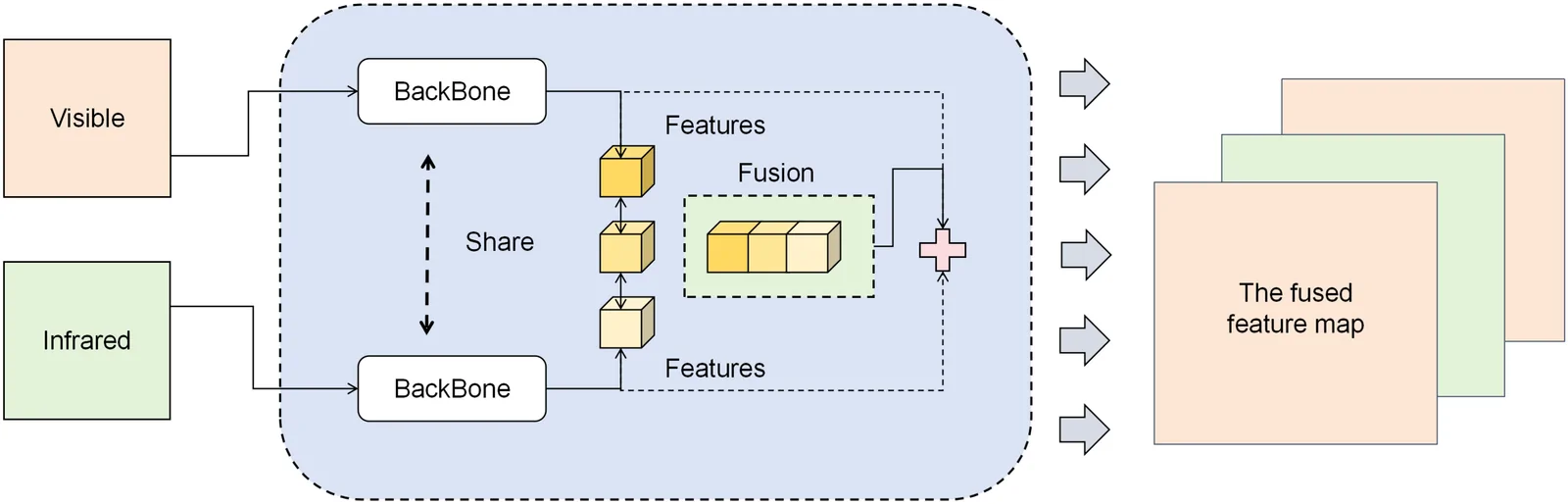
The figure shows the Cross-Modality Guided Dynamic Fusion structure: visible light and infrared inputs are respectively extracted through the weight-sharing Backbone to extract multi-scale features, and then dynamically guided and semantically compensated in the feature domain through the fusion block. The fused features are weighted and integrated to output a unified feature tensor, providing a cross-modal consistent representation for subsequent detection branches.

The Cross-Modality Guided Dynamic Fusion module is designed to achieve fine-grained alignment between visible and infrared modalities at the early stage of feature extraction. The overall model architecture is shown in Fig 2.

Given a visible image ${𝐈}_{v}\;\in \;{\mathbb{R}}^{H\times W\times 3}$ and an infrared image ${𝐈}_{i}\;\in \;{\mathbb{R}}^{H\times W\times 1}$, we first utilize a shared architectural backbone with modality-specific parameters to extract multi-scale semantic features $\{{𝐅}_{v}^{l}{\}}_{l=1}^{L}$ and $\{{𝐅}_{i}^{l}{\}}_{l=1}^{L}$:


$$\begin{array}{c} {𝐅}_{m}^{l}={ℬ}_{m}^{\;l}({𝐈}_{m}),\;m\in \{v,i\},\;l=1,...,L \end{array}$$ (1)

Next, a 1×1 convolution is applied to project both feature maps into a unified channel space, generating modality-specific projected features ${𝐏}_{m}^{l}$ to eliminate dimensional mismatches:


$$\begin{array}{c} {𝐏}_{m}^{l}={𝐖}_{p}^{l}\;*\;{𝐅}_{m}^{l}+{𝐛}_{p}^{l} \end{array}$$ (2)

The projected features from both modalities are concatenated along the channel dimension to form a fusion candidate tensor ${𝐂}^{l}$, which is then passed through global average pooling to obtain a cross-modal context vector ${𝐳}^{l}$:


$$\begin{array}{c} {𝐂}^{l}=[{𝐏}_{v}^{l}\;\|\;{𝐏}_{i}^{l}],\;{𝐳}^{l}=\text{GAP}\;({𝐂}^{l}) \end{array}$$ (3)

The cross-modal dynamic weights $\alpha^{l}\;\in \;[0,1{]}^{C}$ are computed from ${𝐳}^{l}$ via two fully connected layers followed by a Sigmoid activation function, enabling adaptive channel-wise recalibration of visible and infrared contributions:


$$\begin{array}{c} \alpha^{l}=\sigma \;({𝐖}_{2}^{l}\;\delta \;({𝐖}_{1}^{l}{𝐳}^{l})) \end{array}$$ (4)

A weighted summation guided by $\alpha^{l}$ is then performed to explicitly integrate complementary information from both modalities, resulting in the fused feature representation ${𝐅}_{f}^{l}$:


$$\begin{array}{c} {𝐅}_{f}^{l}=\alpha^{l}\;⊙\;{𝐏}_{v}^{l}\;+\;(1-\alpha^{l})⊙{𝐏}_{i}^{l} \end{array}$$ (5)

To capture cross-scale dependencies, CMG-DF further introduces a lightweight context gating mechanism ${𝐆}^{l}$ in each layer to refine ${𝐅}_{f}^{l}$ in a fine-grained manner. This gating mechanism adaptively modulates channel-wise feature responses based on local semantic context, allowing the network to emphasize informative regions while suppressing background noise. By doing so, it enhances the selectivity and expressiveness of the fused features across spatial resolutions:


$$\begin{array}{c} {𝐆}^{l}=\tau \;({\text{Conv}}_{1\times 1}\;({𝐅}_{f}^{l})),\;{\hat{𝐅}}_{f}^{l}={𝐆}^{l}⊙{𝐅}_{f}^{l} \end{array}$$ (6)

Finally, all refined features ${\hat{𝐅}}_{f}^{l}$ across scales are progressively upsampled and concatenated to form the multi-scale fused representation $\tilde{𝐅}$ for subsequent refinement networks:


$$\begin{array}{c} \tilde{𝐅}=\phi ({\hat{𝐅}}_{f}^{1},{\hat{𝐅}}_{f}^{2},...,{\hat{𝐅}}_{f}^{L}) \end{array}$$ (7)

Through the combination of cross-modal dynamic weighting, intra-layer context gating, and cross-layer aggregation, CMG-DF achieves spatiotemporal alignment and semantic compensation between heterogeneous modalities under controllable computational cost, thereby providing a high signal-to-noise and semantically consistent feature foundation for downstream fine-grained small object detection.

### 3.2 Hierarchical scale refinement network

**Fig 3:**
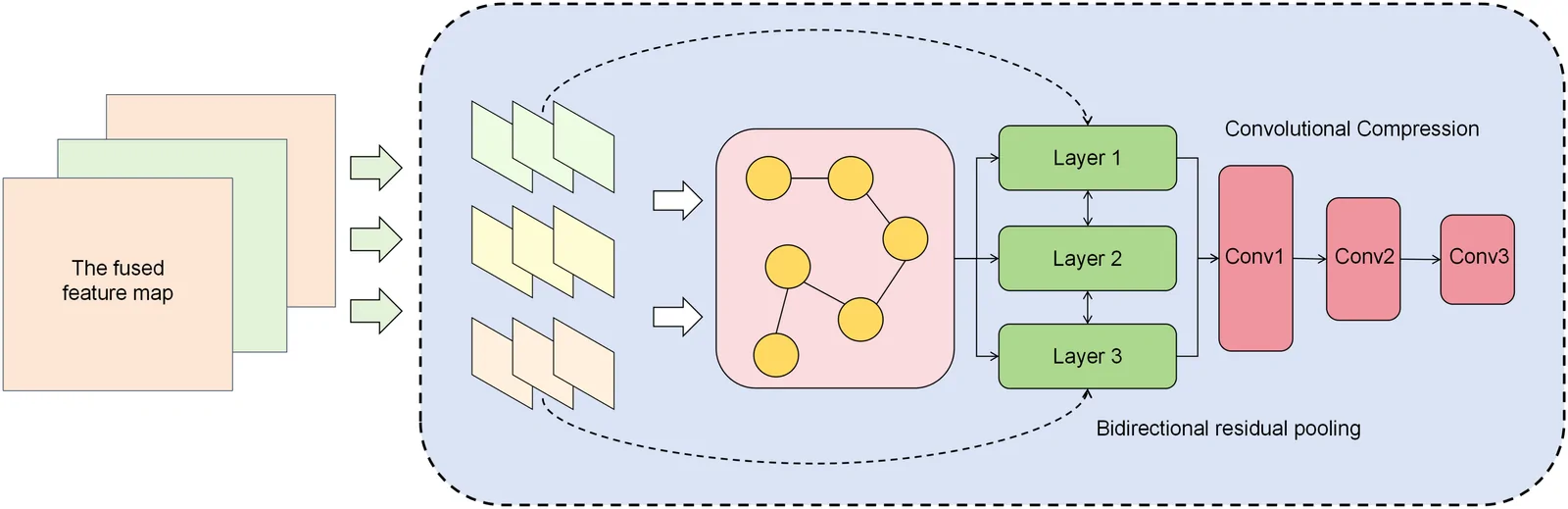
As shown in the figure, the Hierarchical Scale Refinement Network first groups the fused features at multiple scales and associates them with graph convolutions, and then refines them layer by layer through bidirectional residual pooling and convolution compression to output high-resolution and semantically consistent target representations.

After completing cross-modal alignment via the CMG-DF module, the multi-level fused features $\tilde{𝐅}=\{{\tilde{𝐅}}^{1},...,{\tilde{𝐅}}^{L}\}$ still exhibit significant scale disparities and local noise artifacts. To further enhance the discriminability of dense small objects, we propose the *Hierarchical Scale Refinement Network*, which progressively resamples and compensates multi-scale representations through bidirectional “top-down” and “bottom-up” interactions. The network ultimately outputs high-resolution, fine-grained object representations. The overall pipeline consists of four stages: scale grouping, adaptive relational modeling, progressive residual enhancement, and cross-layer fusion, each of which enables deep semantic propagation and local detail recovery while maintaining controlled parameter complexity. Its overall architecture is shown in Fig 3.

We first group the fused features $\tilde{𝐅}$ into *K* resolution levels $𝒮=\{{𝒮}_{1},...,{𝒮}_{K}\}$ based on spatial granularity. For each group, a scale-aware attention vector $\gamma_{k}$ is computed to dynamically modulate the information flow:


$$\begin{array}{c} \gamma_{k}=\sigma \;({𝐖}_{\gamma }^{k}\;\text{GMP}\;({𝒮}_{k})),\;k=1,...,K \end{array}$$ (8)

Next, graph convolution is used to explicitly model intra- and inter-scale dependencies, capturing contextual interactions across resolution levels:


$$\begin{array}{c} {𝐇}_{k}^{\left(t+1\right)}=\psi ({𝐀}_{k}{𝐇}_{k}^{\left(t\right)}{𝐖}_{\theta }^{k}),\;{𝐀}_{k}=\text{softmax}\;({𝐄}_{k}{𝐄}_{k}^{⊤}) \end{array}$$ (9)

where ${𝐄}_{k}$ denotes the embedding matrix and ψ(·) denotes a normalized activation function.

Top-down residual propagation is realized using a learnable scaling factor $\lambda_{k}^{↑}$, allowing high-level semantic downsampling to compensate lower layers:


$$\begin{array}{c} {𝐑}_{k}^{↑}=\lambda_{k}^{↑}\;{\text{Down}}_{2}\;({𝐇}_{k-1}^{\left(T\right)}),\;\lambda_{k}^{↑}=\tanh\;({𝐰}_{k}^{↑}) \end{array}$$ (10)

Symmetrically, bottom-up residual propagation uses a learnable factor $\lambda_{k}^{↓}$ to inject fine-grained details from lower layers into higher semantic levels. This mechanism enables the network to recover spatial structures that are often lost in deep layers due to semantic abstraction, thereby enriching the high-level representations with localized boundary and texture information. By preserving such low-level cues, the model becomes more sensitive to small and occluded targets, which are particularly vulnerable to feature degradation in hierarchical architectures:


$$\begin{array}{c} {𝐑}_{k}^{↓}=\lambda_{k}^{↓}\;{\text{Up}}_{2}\;({𝐇}_{k+1}^{\left(T\right)}),\;\lambda_{k}^{↓}=\tanh\;({𝐰}_{k}^{↓}) \end{array}$$ (11)

The bidirectional residuals are aggregated to form the stage-wise enhanced feature ${\hat{𝐇}}_{k}$:


$$\begin{array}{c} {\hat{𝐇}}_{k}={𝐇}_{k}^{\left(T\right)}+{𝐑}_{k}^{↑}+{𝐑}_{k}^{↓} \end{array}$$ (12)

To suppress redundancy, a channel-wise gating mechanism ${𝐆}_{k}$ is introduced to filter ${\hat{𝐇}}_{k}$ at a fine-grained level:


$$\begin{array}{c} {𝐆}_{k}=\sigma \;({\text{Conv}}_{1\times 1}\;({\hat{𝐇}}_{k})),\;{\tilde{𝐇}}_{k}={𝐆}_{k}⊙{\hat{𝐇}}_{k} \end{array}$$ (13)

Cross-layer fusion is then performed via progressive concatenation and convolutional compression to generate the high-resolution output 𝐔. This fusion strategy aggregates multi-scale representations from different layers, allowing the network to integrate both coarse semantic cues and fine-grained spatial details. The subsequent compression operation serves to harmonize channel dimensions and reduce redundancy, ensuring that the fused features remain compact and computationally efficient:


$$\begin{array}{c} 𝐔=\phi (\text{Concat}[{\tilde{𝐇}}_{1},...,{\tilde{𝐇}}_{K}]) \end{array}$$ (14)

where ϕ(·) denotes a composite operator consisting of Conv 3×3+BN+ReLU layers.

Finally, to adapt the refined features to the detection head, we introduce a scale-balanced mapping η to project 𝐔 into a unified semantic space:


$$\begin{array}{c} {𝐅}_{\text{HSRN}}=\eta \;(𝐔)={\text{Conv}}_{1\times 1}\;(𝐔) \end{array}$$ (15)

With the integration of scale-aware attention, graph-based relational modeling, bidirectional residual compensation, and gated fusion, HSRN progressively refines the cross-modal features produced by CMG-DF. It achieves scale alignment and semantic enhancement across layers, providing noise-suppressed and context-rich representations for subsequent small object detection.

### 3.3 Training goals

To ensure cross-modal consistency of the fused features from CMG–DF, we first introduce a cross-modality consistency loss ${ℒ}_{CM}$, which minimizes the global distributional divergence between the visible and infrared channel attention vectors after fusion. Specifically, it is defined as:


$$\begin{array}{cc} {ℒ}_{CM} & =\frac{1}{K}\sum_{k=1}^{K}\|\underset{\text{Visible Avg}}{\underbrace{\frac{1}{HW}\sum_{x,y}\alpha_{k}(x,y)}}\\ & \;-\underset{\text{Infrared Avg}}{\underbrace{\frac{1}{HW}\sum_{x,y}(1-\alpha_{k}(x,y))}}{\|}_{2}^{2} \end{array}$$ (16)

where $\alpha_{k}$ denotes the dynamic attention weights of the *k*-th channel in CMG–DF, and H×W represents the spatial resolution of the feature map.

To fully exploit the hierarchical scale compensation capability of HSRN, we design a progressive scale consistency loss ${ℒ}_{HS}$, which aligns semantic responses across resolution levels after bidirectional residual propagation:


$$\begin{array}{cc} {ℒ}_{HS} & =\sum_{k=1}^{K-1}{\left\|{𝐑}_{k}^{↑}-{\text{Down}}_{2}\left({𝐑}_{k+1}^{↓}\right)\right\|}_{1}\\ & \;+\sum_{k=2}^{K}{\left\|{𝐑}_{k}^{↓}-{\text{Up}}_{2}\left({𝐑}_{k-1}^{↑}\right)\right\|}_{1} \end{array}$$ (17)

where ${𝐑}_{k}^{↑}$ and ${𝐑}_{k}^{↓}$ denote the top-down and bottom-up residual features, respectively.

For the detection head, we adopt a composite detection loss ${ℒ}_{DET}$ based on the Hungarian matching paradigm of RT–DETR, combining classification and bounding box regression terms:


$$\begin{array}{cc} {ℒ}_{DET} & =\frac{1}{\left|\Omega \right|}\sum_{(i,j)\in \Omega }[\lambda_{cls}\;\underset{\text{Focal Cls}}{\underbrace{-\;\sum_{c}y_{ij}^{c}\log p_{ij}^{c}}}\\ & \;+\;\lambda_{box}\;\underset{\text{L1 Reg}}{\underbrace{\|b_{i}-{\hat{b}}_{j}{\|}_{1}}}\\ & \;+\;\lambda_{giou}\;\underset{\text{GIoU Loss}}{\underbrace{\left(1-GIoU(b_{i},{\hat{b}}_{j})\right)}}] \end{array}$$ (18)

where Ω denotes the set of matched pairs, $y_{ij}^{c}$ and $p_{ij}^{c}$ are the ground-truth label and predicted probability for class *c*, and $b_{i}$, ${\hat{b}}_{j}$ are the ground-truth and predicted bounding boxes, respectively.

Combining the objectives above, the overall training loss is defined as:


$$\begin{array}{c} {ℒ}_{Total}=\lambda_{CM}{ℒ}_{CM}+\lambda_{HS}{ℒ}_{HS}+\lambda_{DET}{ℒ}_{DET}+\lambda_{wd}\sum_{\theta \in 𝒲}\;\|\theta {\|}_{2}^{2} \end{array}$$ (19)

where 𝒲 denotes the set of learnable parameters, and $\lambda_{*}$ are the corresponding loss weights. The total loss is jointly optimized under the RT–DETR framework, effectively leveraging the advantages of CMG–DF and HSRN for cross-modal alignment and scale-aware fine-grained representation learning.

## 4 Dataset and experimental setting

### 4.1 Dataset

#### 4.1.1 SMOD datasets.

The SMOD dataset is a high-quality benchmark for multimodal object detection, recently released by the Department of Automation and the Intelligent Driving Innovation Center at Shanghai Jiao Tong University. Collected using the Asens FV6 binocular platform, SMOD contains 8,676 pairs of precisely aligned visible and infrared images covering a wide range of complex illumination conditions in campus environments. Notably, nighttime scenes account for 38% of the samples, including extreme lighting scenarios such as low illumination and high glare, posing realistic challenges for multimodal perception. The dataset provides dense bounding box annotations for four object categories—pedestrians, riders, vehicles, and bicycles—and introduces four-level occlusion labels (none, slight, moderate, and heavy), enabling fine-grained instance differentiation. Owing to its authentic representation of occlusion, scale variation, and low-light conditions, SMOD serves as a critical testbed for evaluating cross-modal guidance mechanisms and dense small object detection algorithms.

#### 4.1.2 LLVIP datasets.

The LLVIP (Low Light Vision Pedestrian Detection) dataset was released in 2021 as a dedicated benchmark for pedestrian detection under low-light conditions. Jointly developed by the Institute of Automation, Chinese Academy of Sciences, and Peking University, the dataset covers a wide range of indoor and outdoor scenes captured in diverse low-illumination environments. A key characteristic of LLVIP is its complex lighting conditions, with significant illumination variation and noise interference, which pose substantial challenges to the robustness and stability of detection algorithms.

**Fig 4:**
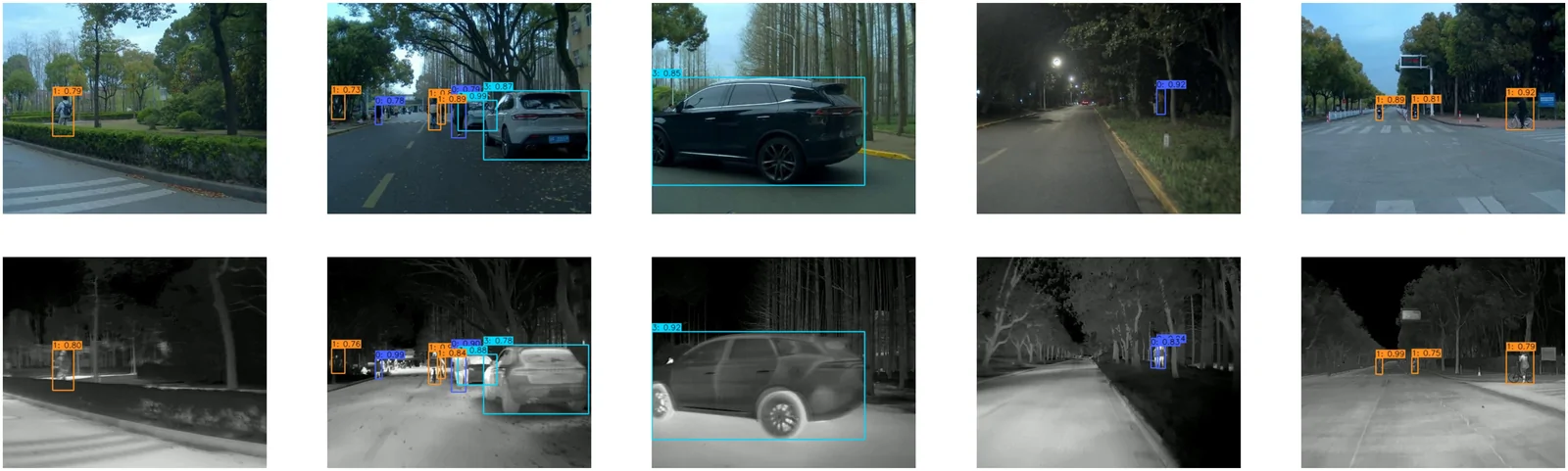
Experimental results on SMOD dataset.

**Fig 5:**
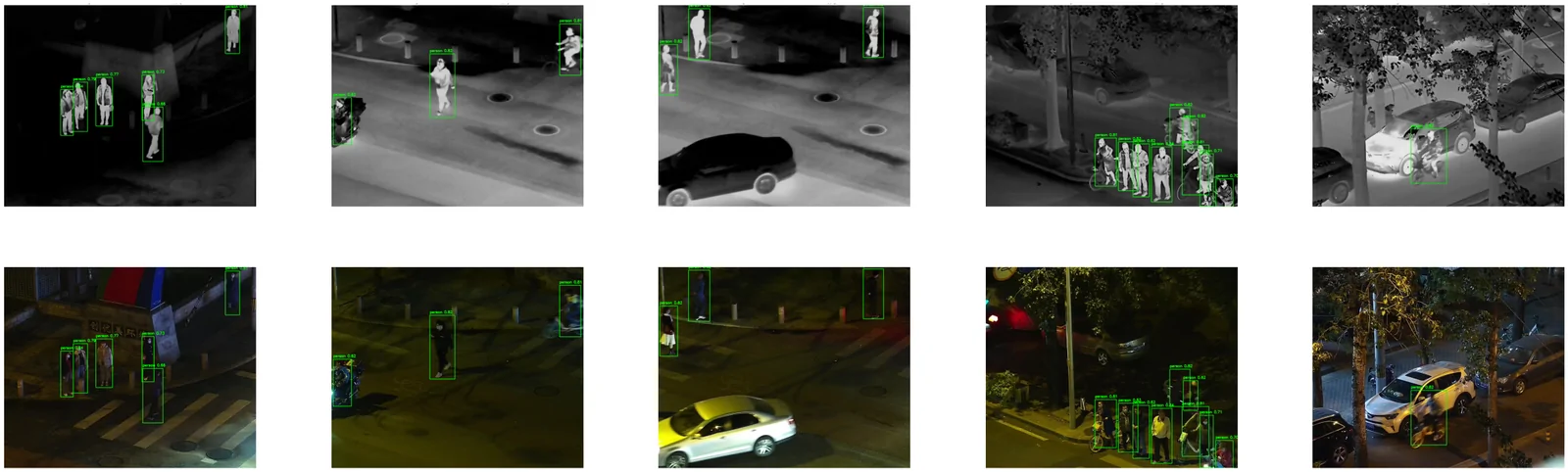
Experimental results on LLVIP dataset.

**Fig 6:**
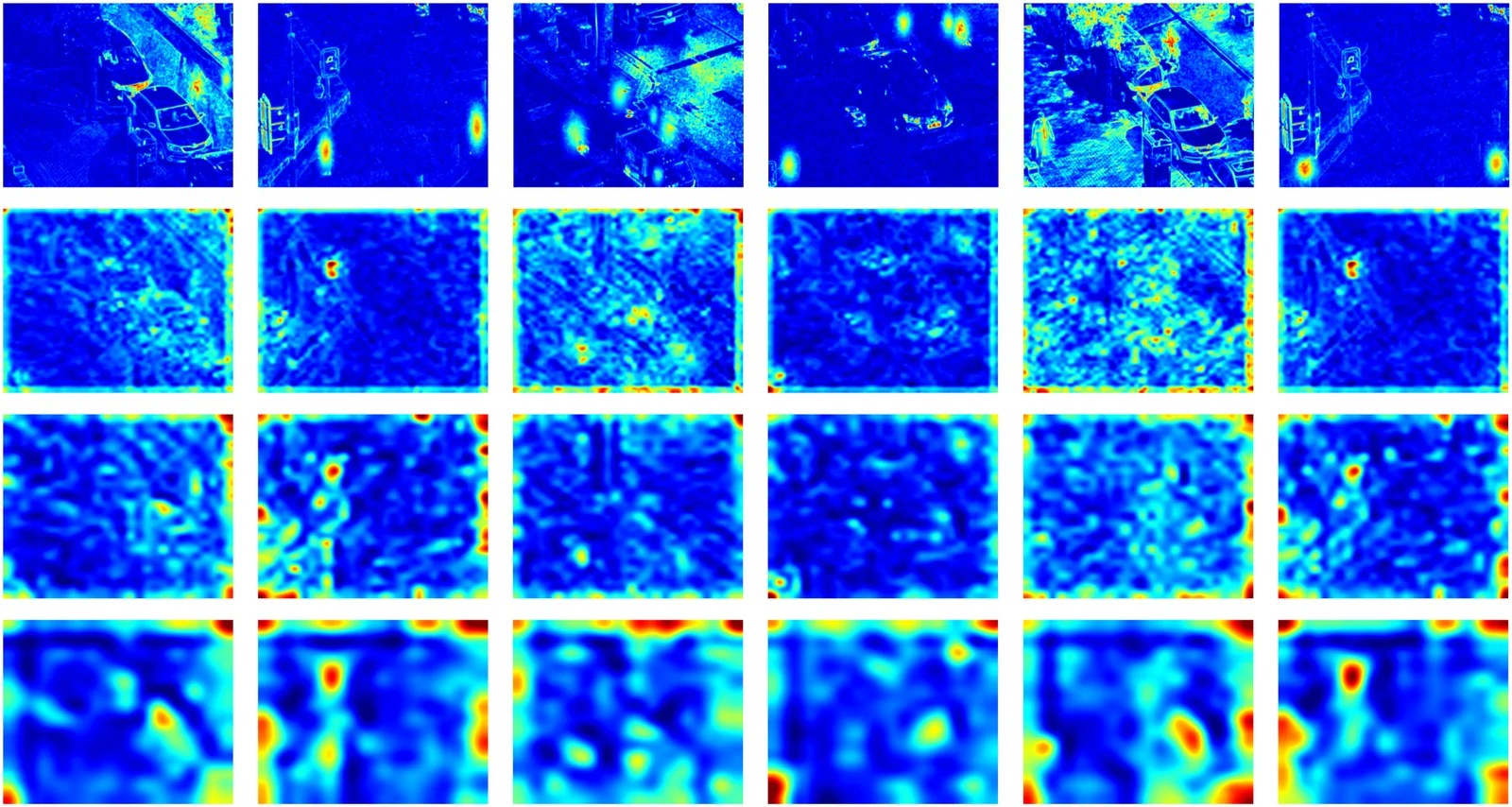
Feature map experimental results.

**Fig 7:**
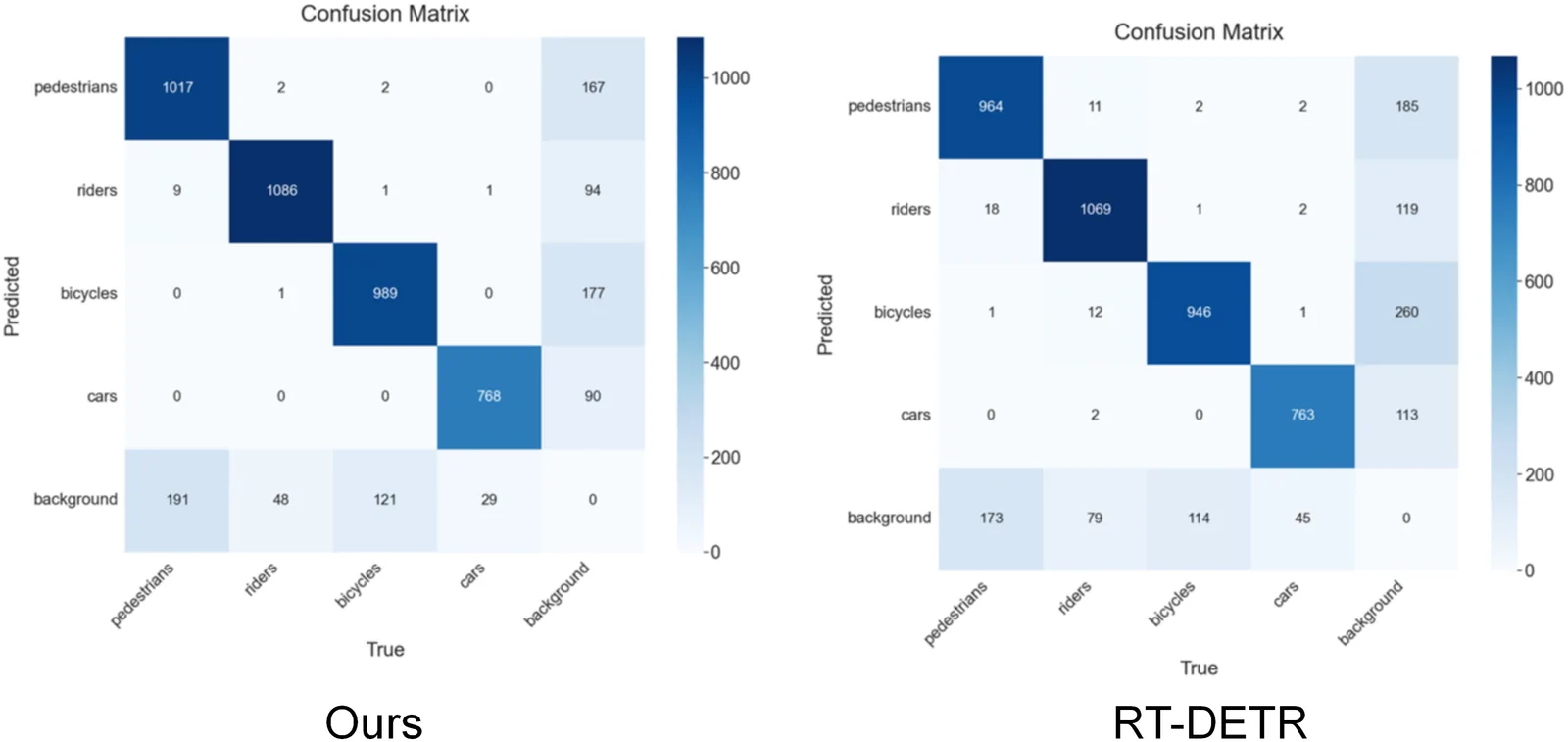
Confusion matrix experiment results.

**Fig 8:**
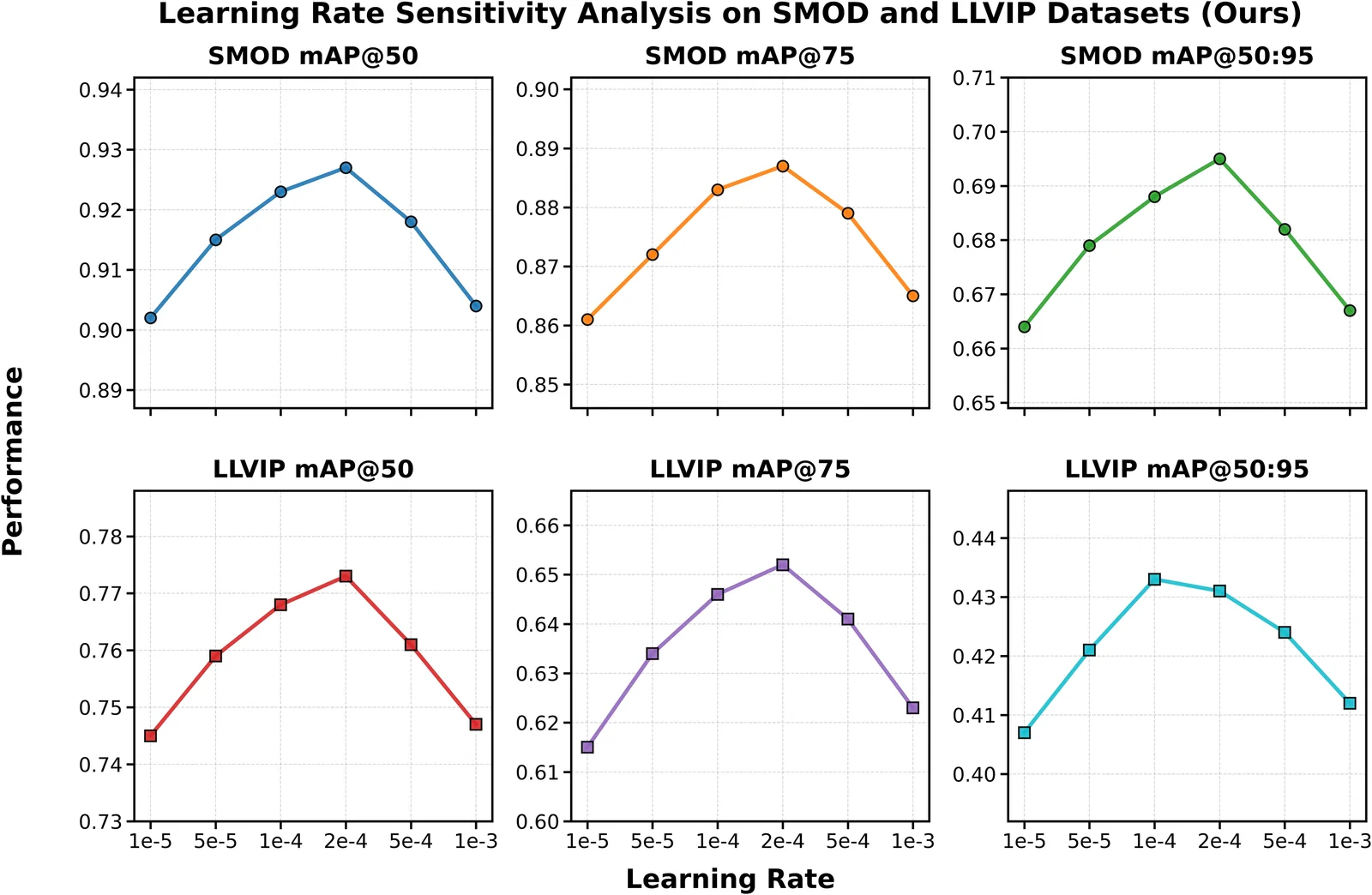
The impact of learning rate on experimental results.

The dataset features high-quality images that offer clearer representations of pedestrian appearance compared to the SMOD dataset, as illustrated in Figs 4–8. As such, LLVIP is widely recognized as one of the most high-quality multispectral pedestrian detection datasets to date. It contains a total of 15,488 pairs of aligned infrared (IR) and RGB images, each with a resolution of 1280×720, and each image includes multiple pedestrian instances.

### 4.2 Experimental setup

In this study, we adopt RT-DETR [34] as the baseline model for algorithmic validation. The proposed CMG-DF and HSRN modules are seamlessly integrated into its main detection pipeline to enable end-to-end training and evaluation. For the backbone, we utilize a ResNet-50 [35] model pretrained on ImageNet-1K [36] to enhance the stability and generalization of feature extraction. All experiments are conducted on a single NVIDIA RTX 2080Ti GPU, with consistent resolution settings maintained during both training and inference phases. The key hyperparameters and hardware configurations are summarized in Table 1.

**Table 1:** Training hyperparameters and hardware configuration.

Component	Setting
Baseline Model	RT-DETR (ResNet-50 backbone)
Backbone Pretraining	ImageNet-1K pretrained
Input Resolution	512 × 512
Batch Size	8
Optimizer	AdamW
Learning Rate	2e-4
Learning Rate Scheduler	Cosine Annealing
Weight Decay	0.05
Warm-up Iterations	500
Training Epochs	50
GPU Device	NVIDIA GeForce RTX 2080Ti
CUDA / cuDNN Version	CUDA 11.3 / cuDNN 8.2
PyTorch Version	2.0
Operating System	Ubuntu 20.04 LTS

## 5 Experimental results and analysis

### 5.1 Comparison of experimental results of other models on different datasets

In the experimental evaluation, we select several mainstream detection frameworks as comparative baselines, including Faster R-CNN [37], YOLOv8 [38], YOLOv10 [39], and YOLOv11 [40], each evaluated under single-modality settings (RGB and IR respectively). To further assess the effectiveness of our proposed approach in multimodal fusion scenarios, we additionally incorporate several representative multimodal detection methods—CFT [41], Super-YOLO [14], GMD-YOLO [42],CMADet [43] and COMO, Hiptrack [44], SHRS-YOLO [45], and DFINet [46]—as comparative benchmarks.

All models are trained and evaluated on the SMOD dataset using identical training and testing splits to ensure fairness. Performance metrics include mAP@50, mAP@75, and mAP@50:95, covering both low and high IoU thresholds. The comparative results are summarized in Table 2.

**Table 2:** Comparative experimental results on the SMOD dataset (%)).

Method	Modality	mAP@50	mAP@75	mAP@50:95	Params	FPS	Inference Time (ms/frame)	GFLOPs	GPU Memory (GB)
Faster R-CNN	RGB	81.3	72.0	54.2	60.2M	26.8	37.31	112.6	3.84
Faster R-CNN	IR	77.6	69.2	51.3	60.2M	26.8	37.31	112.6	3.84
YOLOv8m	RGB	83.9	74.6	56.1	25.9M	92.7	10.79	78.4	2.31
YOLOv8m	IR	79.5	70.3	52.9	25.9M	92.7	10.79	78.4	2.31
YOLOv10m	RGB	86.2	77.4	58.7	15.4M	111.7	8.95	64.7	2.12
YOLOv10m	IR	80.9	72.1	53.4	15.4M	111.7	8.95	64.7	2.12
YOLOv11m	RGB	88.1	79.0	60.2	20.1M	98.5	10.15	69.8	2.18
YOLOv11m	IR	83.7	74.5	55.6	20.1M	98.5	10.15	69.8	2.18
CFT	RGB + IR	90.3	85.1	64.7	67.1M	43.9	22.78	141.5	4.62
Super-YOLO	RGB + IR	91.2	86.5	67.0	47.8M	53.2	18.80	105.3	3.96
GMD-YOLO	RGB + IR	89.5	83.9	63.8	42.3M	55.6	17.99	98.7	3.72
CMADet	RGB + IR	88.7	82.4	62.1	33.3M	72.8	13.74	82.6	3.21
COMO	RGB + IR	90.8	85.7	66.2	20.3M	97.6	10.25	71.4	2.56
Hiptrack	RGB + IR	89.9	84.6	64.9	28.7M	81.4	12.29	88.9	3.08
SHRS-YOLO	RGB + IR	91.5	86.9	67.4	36.8M	69.2	14.45	93.8	3.42
DFINet	RGB + IR	91.0	86.1	66.8	39.5M	62.7	15.95	96.1	3.55
OA-DG [47]	RGB + IR	90.6	85.4	65.8	44.7M	52.6	19.01	102.4	3.89
CDSD [48]	RGB + IR	91.1	86.3	66.9	46.1M	50.8	19.69	108.7	4.05
**Ours**	**RGB + IR**	**92.7**	**88.7**	**69.5**	43.2M	54.1	18.48	101.6	3.78

As shown in Table 2, our proposed method achieves the best performance across all evaluation metrics on the SMOD dataset. In particular, it significantly outperforms other multimodal approaches in terms of mAP@50:95, demonstrating superior fine-grained detection capabilities. Compared to existing multimodal baselines that rely on early or static fusion strategies (e.g., CFT, Super-YOLO, and GMD-YOLO), our CMG-DF module introduces a cross-modality guided mechanism that dynamically recalibrates RGB and IR features in both spatial and semantic dimensions. This leads to enhanced modality responses in key regions and effectively alleviates the feature conflict caused by cross-modal inconsistency.

Moreover, the proposed HSRN module exhibits clear advantages in multi-scale small object modeling through its hierarchical residual guidance and progressive scale refinement mechanism. By constructing a bidirectional residual information flow between high-level and low-level features, HSRN enables semantic-texture compensation and preserves the spatial structures of small objects that are often compressed in deep layers. In addition, the graph-based relational modeling enhances intra-scale contextual consistency, allowing the model to better capture transition boundaries among dense targets.

The performance gap observed between single-modality detectors such as YOLOv8, YOLOv10, and YOLOv11 under RGB and IR conditions further underscores the importance of effective multimodal fusion strategies in complex environments. Our method addresses this challenge by incorporating dynamic weight control and channel-guided modulation in the fusion stage, moving beyond simple concatenation or additive operations. This enables structural guidance of information flow and fine-grained modulation of modality contributions, effectively realizing a form of “modality-adaptive harmonization” for semantic enhancement. The experimental results of LLVIP are further given, and are shown in Table 3.

**Table 3:** Performance comparison on LLVIP dataset under different modality settings (%).

Method	Modality	mAP@50	mAP@75	mAP@50:95	Params	FPS	Inference Time (ms/frame)	GFLOPs	GPU Memory Usage (GB)
Faster R-CNN	RGB	65.2	52.4	32.1	60.2M	26.8	36.80	112.6	3.84
Faster R-CNN	IR	60.8	49.1	29.7	60.2M	26.8	36.84	112.6	3.84
YOLOv8m	RGB	68.6	54.9	34.5	25.9M	92.7	10.83	78.4	2.31
YOLOv8m	IR	64.1	50.5	31.2	25.9M	92.7	11.04	78.4	2.31
YOLOv10m	RGB	71.4	58.0	36.3	15.4M	111.7	8.90	64.7	2.12
YOLOv10m	IR	66.9	53.7	33.0	15.4M	111.7	8.87	64.7	2.12
YOLOv11m	RGB	73.1	60.8	38.4	20.1M	98.5	10.29	69.8	2.18
YOLOv11m	IR	68.5	55.2	34.6	20.1M	98.5	10.35	69.8	2.18
CFT	RGB + IR	74.6	61.9	39.7	67.1M	43.9	22.93	141.5	4.62
Super-YOLO	RGB + IR	75.8	63.3	41.5	47.8M	53.2	18.60	105.3	3.96
GMD-YOLO	RGB + IR	73.9	60.2	38.9	42.3M	55.6	17.83	98.7	3.72
CMADet	RGB + IR	72.7	59.4	37.8	33.3M	72.8	13.60	82.6	3.21
COMO	RGB + IR	76.4	64.1	42.3	20.3M	97.6	10.17	71.4	2.56
Hiptrack	RGB + IR	75.6	62.8	41.2	28.7M	81.4	12.27	88.9	3.08
SHRS-YOLO	RGB + IR	74.9	61.5	40.4	36.8M	69.2	14.63	93.8	3.42
DFINet	RGB + IR	76.1	63.7	42.0	39.5M	62.7	16.24	96.1	3.55
OA-DG	RGB + IR	75.7	63.0	41.4	44.7M	52.6	18.90	102.4	3.89
CDSD	RGB + IR	76.2	63.8	42.0	46.1M	50.8	19.99	108.7	4.05
**Ours**	**RGB + IR**	**77.3**	**65.2**	**43.1**	43.2M	54.1	18.83	101.6	3.78

These experimental results further validate the robustness and generalization capability of the proposed cross-modal dynamic fusion and hierarchical progressive modeling mechanisms under low-light and multimodal conditions. The CMG-DF module effectively addresses the pronounced response discrepancy between infrared and visible modalities in the LLVIP dataset by dynamically guiding modality-specific weights. Meanwhile, the HSRN module demonstrates strong performance in handling blurred targets and scale variance in complex scenes. By leveraging bidirectional residual compensation and enhanced contextual modeling, it enables accurate recovery of weak object boundaries. The synergistic integration of these two components allows the model to maintain superior detection performance even in challenging nighttime scenarios.

### 5.2 Ablation model experimental results and analysis

Following the comparison with mainstream detection methods, we further conduct a series of component-level ablation studies on both the SMOD and LLVIP datasets to more systematically evaluate the individual and joint contributions of the proposed CMG-DF and HSRN modules. In addition to the overall module-level ablations, we explicitly examine the effect of the dynamic attention weighting mechanism in CMG-DF by replacing the learnable attention weights with fixed weights. For HSRN, the respective contributions of scale-aware attention and graph-based relational modeling are separately evaluated by removing each component while retaining the remaining structure. The same ablation protocol is further repeated on the LLVIP dataset to examine whether these design choices remain effective under a different data distribution. The corresponding results are presented in Tables 4 and 5.

**Table 4:** Ablation study of key components on SMOD dataset (%).

Setting	CMG-DF	HSRN	mAP@50	mAP@75	mAP@50:95
**Baseline**	–	–	88.5	78.2	60.4
**+CMG-DF**	✓	–	90.1	80.6	63.0
**+HSRN**	–	✓	89.3	79.4	61.5
**Ours**	✓	✓	**92.7**	**88.7**	**69.5**
**Ablation on CMG-DF**					
static fusion	✓	–	88.9	79.1	61.8
fixed attention weights	✓	–	89.5	79.8	62.2
**Ablation on HSRN**					
single-stage refinement	–	✓	88.7	78.9	60.9
w/o multi-resolution context aggregation	–	✓	89.1	79.0	61.3
w/o feature resampling/alignment	–	✓	88.5	78.6	60.7
w/o scale-aware attention	–	✓	88.9	79.0	61.0
w/o graph-based relational modeling	–	✓	88.8	78.8	60.8

**Table 5:** Ablation study of key components on LLVIP dataset (%).

Setting	CMG-DF	HSRN	mAP@50	mAP@75	mAP@50:95
**Baseline**	–	–	72.9	59.1	37.2
**+CMG-DF**	✓	–	75.0	62.1	40.1
**+HSRN**	–	✓	74.0	60.5	38.6
**Ours**	✓	✓	**77.3**	**65.2**	**43.1**
**Ablation on CMG-DF**					
static fusion	✓	–	73.8	60.3	38.6
fixed attention weights	✓	–	74.2	61.0	39.1
**Ablation on HSRN**					
single-stage refinement	–	✓	73.4	59.8	37.9
w/o multi-resolution context aggregation	–	✓	73.8	60.2	38.3
w/o feature resampling/alignment	–	✓	73.1	59.4	37.5
w/o scale-aware attention	–	✓	73.6	60.0	38.1
w/o graph-based relational modeling	–	✓	73.5	59.9	38.0

As shown in Tables 4 and 5, both CMG-DF and HSRN provide consistent performance improvements over the baseline on the two datasets, while their joint use achieves the best results across all three evaluation metrics. On SMOD, introducing CMG-DF improves mAP@50, mAP@75, and mAP@50:95 from 88.5%, 78.2%, and 60.4% to 90.1%, 80.6%, and 63.0%, respectively. Replacing the learnable dynamic attention weights with fixed weights reduces these values to 89.5%, 79.8%, and 62.2%, corresponding to decreases of 0.6, 0.8, and 0.8 percentage points. A larger reduction is observed when dynamic fusion is replaced by static fusion. A similar trend is observed on LLVIP, where fixed attention weights decrease mAP@50, mAP@75, and mAP@50:95 by 0.8, 1.1, and 1.0 percentage points relative to the complete CMG-DF configuration. These results indicate that the improvement of CMG-DF is not solely attributable to multimodal feature aggregation, but is also associated with the adaptive modulation of modality contributions through learnable dynamic attention weights.

The component-level results of HSRN further clarify the contributions of its internal scale modeling mechanisms. On SMOD, removing scale-aware attention decreases mAP@50:95 from 61.5% to 61.0%, while removing graph-based relational modeling further reduces it to 60.8%. Corresponding decreases are also observed for mAP@50 and mAP@75. On LLVIP, removing scale-aware attention decreases mAP@50:95 from 38.6% to 38.1%, whereas removing graph-based relational modeling results in 38.0%. The single-stage refinement, multi-resolution context aggregation, and feature resampling/alignment ablations exhibit similar performance degradation on both datasets. Importantly, the overall trends of the component ablations remain consistent between SMOD and LLVIP despite their different scene characteristics and data distributions. These results provide additional evidence that dynamic cross-modal weighting in CMG-DF and hierarchical scale-aware relational modeling in HSRN contribute consistently to the detection performance of the proposed framework rather than being specific to a single dataset.

### 5.3 Visualizing experimental results

In the qualitative experiments, we further present intuitive detection results on the SMOD and LLVIP datasets to demonstrate the practical performance of the proposed method in dense small object scenarios. Additionally, we visualize the feature activation maps from the ablation modules to analyze the advantages of CMG-DF and HSRN in feature enhancement and semantic focus. Furthermore, we incorporate confusion matrix visualizations to quantitatively assess the model’s ability to distinguish between different object categories. These multi-perspective analyses collectively verify the effectiveness and robustness of our algorithm under complex multimodal conditions.

#### 5.3.1 Visual detection examples.

This paper first gives the intuitive detection results of the SMOD dataset, and the experimental results are shown in Fig 4.

The qualitative results further demonstrate the practical detection performance of our method on the SMOD dataset, where the model consistently identifies small, distant, or partially occluded targets even under challenging conditions such as complex lighting and strong background interference. The comparison between RGB and IR modalities highlights their complementary characteristics: infrared imagery provides clearer thermal boundary cues in nighttime or low-light scenarios, while RGB images offer richer semantic structure for scene understanding. Benefiting from this complementarity, the proposed CMG-DF module dynamically adjusts modality weights to adaptively enhance key object regions across diverse scenes while suppressing irrelevant background noise, enabling tight bounding boxes and stable category discrimination in crowded vehicle areas and pedestrians near image borders. This robustness is further supported by the bidirectional residual guidance mechanism in HSRN, which promotes semantic consistency across scales and strengthens contextual compensation in ambiguous regions; notably, the infrared modality also exhibits stronger localization sensitivity, improving overall multimodal fusion robustness. In addition, the confidence distribution of predicted bounding boxes remains highly concentrated, indicating the model’s strong ability to prioritize salient targets under different modal conditions, and overall these observations confirm that the joint mechanism of cross-modality guidance and hierarchical refinement enhances consistent feature modeling and object discrimination across heterogeneous modalities, demonstrating strong applicability and generalization in complex real-world environments.

Furthermore, the experimental results of LLVIP are given, and the experimental results are shown in Fig 5.

In the qualitative results on the LLVIP dataset, the proposed method demonstrates stable detection capability for weak targets such as pedestrians and vehicles under nighttime low-illumination and cross-modal interference conditions. By leveraging the CMG-DF module for dynamic fusion of RGB and IR features, the model effectively enhances the discriminability of blurred regions in low-light imagery. Simultaneously, the HSRN module preserves and refines structural details across different object scales, enabling the model to maintain highly consistent bounding boxes even in scenarios where object boundaries are vague or semantic cues are incomplete.

These results indicate that the proposed model exhibits strong generalization in both cross-modal fusion and multi-scale representation learning. In particular, it is well suited for dense small object recognition tasks in complex visual perception environments, where robustness to illumination variation and modality inconsistency is essential.

#### 5.3.2 Experimental results of different modal characteristic diagrams.

We further present the model’s response distribution across different hierarchical feature spaces to reveal the internal flow and aggregation of semantic information within the network. By visualizing feature maps at several key stages, we can intuitively observe the enhanced saliency and structural preservation of small object regions. This analysis facilitates a mechanism-level understanding of the proposed modules, highlighting their contributions to feature alignment, scale compensation, and semantic focusing. It also provides interpretability evidence that supports the rationality and effectiveness of the overall model design. The experimental results are shown in Fig 6.

From the hierarchical feature map visualizations, our method shows strong edge-response capability even in early shallow layers, enabling preliminary localization of object structural contours, while deeper layers progressively concentrate activations on semantically salient regions, forming a smooth transition from spatial details to high-level semantics. With the CMG-DF dynamic fusion mechanism, this layered representation effectively captures the spatial and semantic coupling between modalities and reinforces perceptual consistency over target-relevant regions. Moreover, under nighttime or low-light conditions, the infrared modality contributes clear thermal responses in higher-level features, whereas the visible modality provides richer textures and boundary cues in intermediate layers; by further integrating bidirectional residual paths and scale-aware attention modeling in HSRN, semantic abstraction and scale adaptation are jointly optimized across layers, yielding more discriminative object representations at the final fusion stage and improving the model’s focus on weak and small targets while enhancing structural robustness under complex multimodal conditions.

#### 5.3.3 Confusion matrix comparison experiment results.

Finally, this paper gives the experimental results of the confusion matrix using the SMOD dataset as an example, and the experimental results are shown in Fig 7.

The confusion matrix comparison reveals that our method significantly reduces the misclassification rate to background for major object categories such as *pedestrians*, *riders*, and *bicycles*, compared to RT-DETR. This indicates that the model exhibits stronger recognition capability for weak targets in complex scenes. This advantage stems from the CMG-DF module, which dynamically guides the fusion of RGB and infrared features, thereby enhancing semantic consistency across modalities in small-object regions. As a result, the model is able to maintain focus on true targets even under strong background interference.

Moreover, the HSRN module incorporates multi-scale residual information flow and context-aware modeling, enabling accurate category discrimination even under challenging conditions such as blurred boundaries or occlusions. This significantly mitigates inter-class confusion. In contrast to RT-DETR’s high false positive rate between *bicycles* and *background*, our model demonstrates stronger structural awareness and boundary separation capability. These results further validate that the proposed method improves both intra-class consistency and inter-class separability in multimodal small object detection tasks.

### 5.4 Hyperparameter sensitivity experiment results

This paper also gives the experimental results of the impact of learning rate on two data sets, and the experimental results are shown in Fig 8.

From the experimental results, it can be observed that the learning rate has a significant impact on the model’s performance in multimodal dense small object detection tasks. When the learning rate increases from $1\times 10^{-5}$ to $2\times 10^{-4}$, all metrics (mAP@50, mAP@75, and mAP@50:95) show an upward trend. This indicates that a moderate learning rate helps stabilize the optimization of cross-modal feature fusion and scale refinement, allowing the model to better capture the complementary information between infrared and visible-light features. However, when the learning rate continues to increase to $5\times 10^{-4}$ or $1\times 10^{-3}$, the performance declines. This suggests that an excessively large learning rate disrupts modal alignment and semantic consistency, leading to unstable training and the loss of fine-grained target features. Overall, the optimal learning rate is approximately $2\times 10^{-4}$, where the model achieves peak performance on both the SMOD and LLVIP datasets, confirming that the proposed cross-modal guidance and hierarchical scale refinement mechanisms can fully demonstrate robust detection capability under an appropriate learning rate.

**Fig 9:**
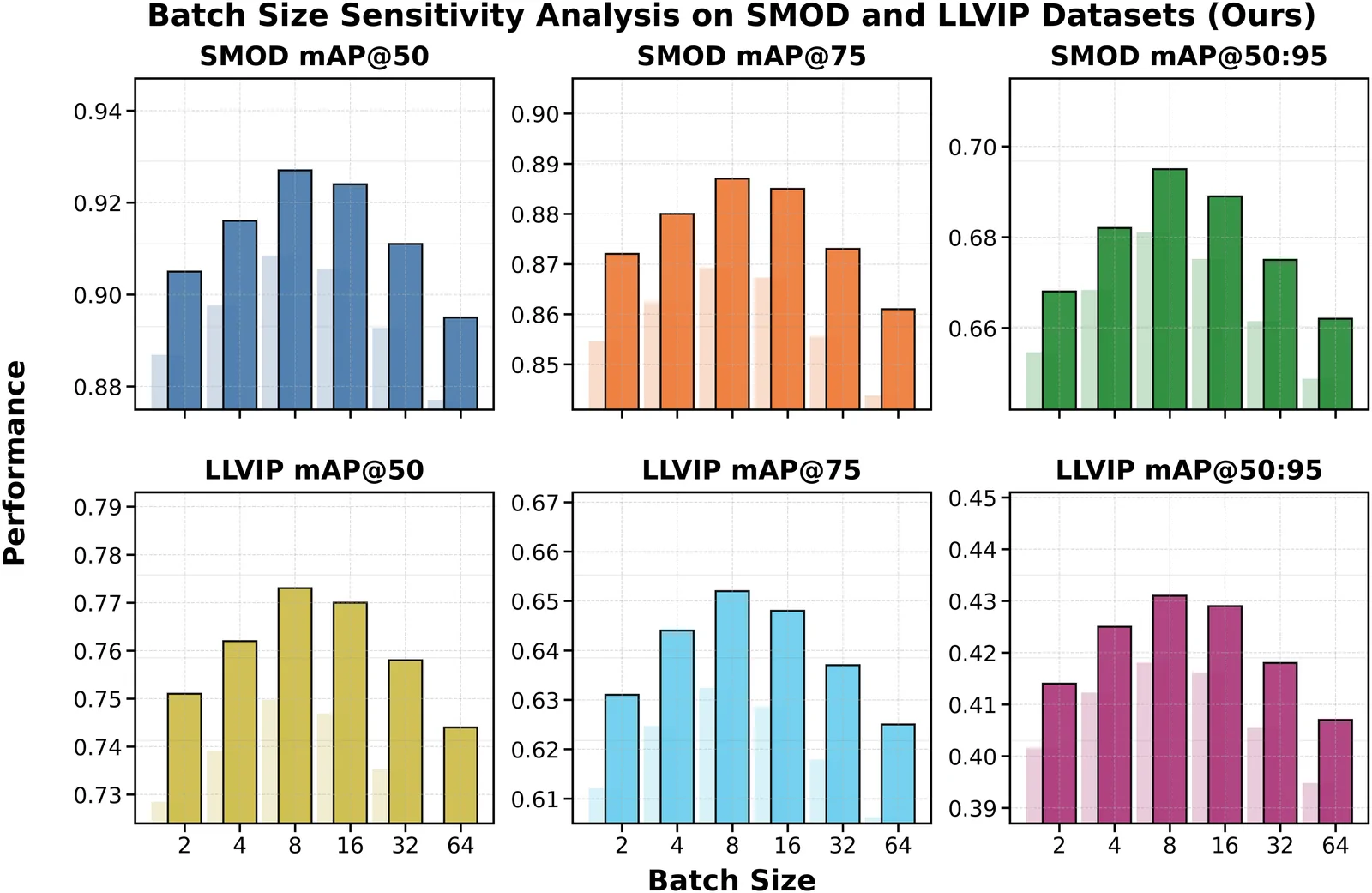
The impact of batch size on experimental results.

This paper also gives the impact of Batch Size on the experimental results, and the experimental results are shown in Fig 9.

From the experimental results, it can be seen that the batch size has a clear impact on the model’s performance. When the batch size increases from 2 to 8, the mAP@50, mAP@75, and mAP@50:95 on the SMOD and LLVIP datasets show a continuous upward trend. This indicates that a moderate batch size can enhance the effectiveness of cross-modal feature fusion while maintaining stable gradient updates, making the model more robust in small object detection and modality alignment. When the batch size further increases to 32 or 64, the performance declines, suggesting that an excessively large batch size reduces the sensitivity of gradient updates and smooths the feature distribution, which weakens the model’s ability to capture fine-grained semantics and small-scale targets. Overall, a batch size of 8 achieves the best balance between computational efficiency and detection accuracy, confirming that the proposed cross-modal guidance and hierarchical scale refinement mechanisms can stably exploit multimodal fusion advantages under a reasonable training scale.

### 5.5 Comparison with Simple Fusion Baselines

To better justify the design choices and additional complexity of the proposed CMG-DF module, we compare it with several widely used lightweight fusion baselines commonly adopted in multimodal detection. Specifically, we include straightforward feature concatenation as well as representative attention-based recalibration strategies to examine whether simple fusion mechanisms are sufficient to handle modality discrepancy in dense small-object scenarios. The experimental results are shown in Table 6.

**Table 6:** Comparison with simple fusion baselines on SMOD and LLVIP datasets.

SMOD dataset				LLVIP dataset			
Method	mAP@50	mAP@75	mAP@50:95	Method	mAP@50	mAP@75	mAP@50:95
Concat	85.4	74.1	55.8	Concat	68.2	52.7	33.5
SE	88.9	78.3	61.2	SE	72.4	58.3	37.9
CBAM	90.7	82.5	65.1	CBAM	75.1	61.2	40.6
Ours	92.7	88.7	69.5	Ours	77.3	65.2	43.1

As shown in Table 6, our proposed fusion strategy consistently outperforms straightforward concatenation and representative lightweight attention-based baselines on both SMOD and LLVIP. Notably, the improvements are more pronounced under stricter localization criteria, indicating that the proposed cross-modality guided fusion not only boosts overall detection accuracy but also yields more precise bounding-box localization in dense small-object scenarios across heterogeneous modalities.

### 5.6 Experimental results at different resolutions

To further evaluate the influence of input resolution on detection performance, we conduct additional experiments using four input resolutions, including 320×320, 416×416, 512×512, and 640×640. The same evaluation protocol is applied to both the SMOD and LLVIP datasets, and the corresponding results are reported in Tables 7 and 8.

**Table 7:** Detection performance of the proposed method under different input resolutions on the SMOD dataset (%).

Input Resolution	mAP@50	mAP@75	mAP@50:95
320×320	87.8	84.4	64.5
416×416	90.3	86.5	67.6
512×512	92.7	88.7	69.5
640×640	93.3	89.9	70.8

**Table 8:** Detection performance of the proposed method under different input resolutions on the LLVIP dataset (%).

Input Resolution	mAP@50	mAP@75	mAP@50:95
320×320	74.8	60.7	37.2
416×416	75.9	63.3	41.7
512×512	77.3	65.2	43.1
640×640	77.6	66.7	44.2

As shown in Tables 7 and 8, increasing the input resolution consistently improves detection performance on both datasets. On SMOD, mAP@50:95 increases from 64.5% at 320×320 to 70.8% at 640×640, while on LLVIP it increases from 37.2% to 44.2%. The improvement becomes relatively smaller when the resolution increases from 512×512 to 640×640, particularly for mAP@50, indicating a gradually diminishing performance gain at higher resolutions. These results show that input resolution has a measurable influence on detection accuracy across both datasets, with higher resolutions generally providing better detection performance under the current experimental settings.

## 6 Limitation

Although the proposed method demonstrates competitive performance on two public multimodal detection benchmarks, this study mainly focuses on comparisons with mainstream single-modality detectors, representative multimodal detection methods, recent detection-related baselines, and simple fusion strategies. Comparisons with domain generalization and domain adaptation methods are still limited. Since multimodal dense small object detection may be affected by scene shifts, illumination changes, sensor differences, and cross-dataset distribution gaps, future work will further incorporate domain generalization and adaptation baselines to more comprehensively evaluate the robustness and transferability of the proposed framework under unseen environments.

In addition, the current experimental analysis mainly reports overall detection metrics, including mAP@50, mAP@75, mAP@50:95, Params, and FPS. Statistical significance testing, confidence interval estimation, and fine-grained attribute-specific analysis under challenging conditions such as scale variation, occlusion, low illumination, and background clutter are not yet fully explored. Future work will further introduce statistical testing and attribute-level evaluation to more rigorously verify where the proposed method achieves the most significant improvements and to provide a more comprehensive understanding of its robustness in complex multimodal detection scenarios.

## 7 Conclusion

This paper addresses the challenges of modality heterogeneity, scale variation, and background interference in dense small object detection under multimodal conditions. We propose a novel detection framework that integrates cross-modality guidance with hierarchical scale refinement. The CMG-DF module enables dynamic fusion and adaptive enhancement of visible and infrared features, while the HSRN module ensures multi-scale semantic consistency and context-aware residual compensation. Together, these components significantly enhance the model’s robustness in detecting weak targets under complex environments. Extensive experiments on multiple public multimodal datasets demonstrate that our approach outperforms existing mainstream methods in terms of accuracy, stability, and qualitative interpretability, showcasing strong generalization and practical applicability.

In future work, we plan to explore the integration of cross-modal semantic guidance with temporal perception strategies to enhance the model’s capability in dynamic video-based object detection and behavior analysis. We will also extend the proposed framework to additional sensing modalities to validate its flexibility in broader multimodal perception settings. To improve robustness across diverse scenes and cross-city distributions, we intend to investigate domain generalization and adaptation strategies that better handle scene-level shifts and environmental variations. Additionally, to address the computational overhead associated with high-resolution image processing, we aim to incorporate more efficient modality compression and attention reconstruction modules, and study scalable training/inference pipelines to facilitate real-world deployment under resource-constrained or real-time requirements, promoting the advancement of multimodal small object detection towards real-time and lightweight deployment.
